# Editorial: Discovery of small molecule lead compounds: a driving force to unravel new anti-cancer targets and mechanisms, volume III

**DOI:** 10.3389/fonc.2026.1875555

**Published:** 2026-05-27

**Authors:** Yan Zhang, Leilei Fu, Yinu Wang

**Affiliations:** 1 School of Traditional Chinese Materia Medica, Shenyang Pharmaceutical University, Shenyang, China; 2 School of Life Science and Engineering, Southwest Jiaotong University, Chengdu, China; 3 Feinberg School of Medicine, Northwestern University, Chicago, IL, United States

**Keywords:** computational biology, drug resistance, lead compounds, molecular targets, natural products

Natural products featuring complex structures and a wide range of biological activities serve as vital sources for the development of novel anticancer agents, particularly due to their capacity to overcome multi-drug resistance. An effective strategy for discovering new anticancer drugs involves seeking lead compounds that target previously unexplored molecular entities, including specific enzymes, receptors, and genes. Advanced approaches such as computational biology and high-throughput screening can further aid in identifying promising drug candidates. This Research Topic highlights recent advances in this area and comprises eight articles. We had the opportunity to review a broad spectrum of both original research and review articles within the field. In the following, we present a synthesis of the key findings and concepts elaborated in each of the accepted contributions.

Liu et al. uncovered a novel mechanism by which parthenolide (PTL), a natural compound from traditional Chinese medicine, exerts its anti−lung adenocarcinoma (LUAD) effects. Through integrated proteomic, metabolomic, and computational analyses, PTL was found to significantly modulate amino acid metabolism and oxidative stress responses in LUAD cells, with GCTG identified as a key therapeutic target. Both *in vitro* and *in vivo* validations confirmed that PTL targeting of GCTG inhibits tumor cell proliferation and alters metabolic and redox states. Collectively, these findings not only provide new insights into the anti−LUAD action of PTL but also establish GCTG as a promising molecular target, thereby laying a critical foundation for future drug development and therapeutic strategies.

In this study, Du et al. revealed the therapeutic potential of wogonin against prolactinoma and its underlying mechanisms. Through network pharmacology, molecular docking, and experimental validation, the research identifies key hub targets including EGFR, BCL2, and PTGS2. Notably, wogonin suppresses prolactinoma cell proliferation and induces apoptosis via inhibition of the PI3K/AKT signaling pathway. Furthermore, wogonin not only inhibits tumor growth but also enhances sensitivity to bromocriptine. These findings establish wogonin as a promising multi-target agent for prolactinoma and provide a strong foundation for future drug development.

In the review, Chen et al. focused on the challenge of drug resistance in breast cancer treatment. While existing inhibitors like tamoxifen have improved outcomes, resistance remains a major obstacle. The article evaluates current resistance mechanisms and highlights emerging therapeutic targets, including cell cycle checkpoint molecules, cancer stem cell-related factors, and anti-apoptotic proteins. Corresponding small-molecule inhibitors have shown promise in overcoming resistance in preclinical and clinical studies. Collectively, this review provides critical insights and innovative strategies to guide future development of therapies for drug-resistant breast cancer.

Wang et al. successfully identified two novel VEGFR-2 inhibitors, 17.3.1.7.8 and BMC_0005, from the African natural product database (AfroDb) as promising therapeutic candidates for papillary thyroid carcinoma (PTC). Through computational approaches including virtual screening and molecular dynamics simulations, both compounds demonstrated strong and stable binding to VEGFR-2, along with favorable drug-like properties. These findings highlight these natural-derived compounds as valuable leads for VEGFR-2 targeted therapy, providing a solid foundation for further preclinical development against PTC.

This study demonstrates that lorlatinib, a third−generation ALK inhibitor, may be effective against a broad range of ALK mutations beyond non−small cell lung cancer. By analyzing cancer genomic data and using molecular docking, Zapata Dongo et al. found that lorlatinib consistently binds strongly to multiple deleterious ALK variants. These findings highlight the potential of repurposing lorlatinib for a wider spectrum of ALK−positive tumors and underscore the value of computational approaches in expanding cancer therapeutic options.

Shahnawaz Khan et al. identified radotinib and alectinib as promising repurposed candidates for targeting MEK1 in cancer therapy. Through computational screening and simulations, both compounds demonstrated superior binding stability to MEK1 compared to existing inhibitors. These findings suggest that radotinib and alectinib may help overcome the toxicity and resistance issues associated with current MEK1-targeted treatments, offering a cost-effective and rapid therapeutic alternative. Experimental validation is still needed to confirm their clinical potential.

Valenzuela-Valderrama et al. developed predictive 3D-QSAR models for synthetic chalcone derivatives as potential treatments for ovarian cancer, including chemoresistant forms. The models successfully guided the identification of active chalcones that exhibited strong antiproliferative effects in both standard and cisplatin-resistant ovarian cancer cells. Mechanistically, these compounds act through a ROS-dependent pathway. These findings highlight certain chalcone derivatives as promising candidates for overcoming chemoresistance in ovarian cancer therapy.

This review highlights the potential of steroidal saponins (SSs) as natural modulators of the PI3K/Akt pathway for cancer therapy. SSs exert anticancer effects through multiple mechanisms, including inducing apoptosis, inhibiting metastasis and angiogenesis, reversing multidrug resistance, and modulating the tumor microenvironment. However, clinical translation remains limited due to challenges such as low bioavailability, systemic toxicity, and poor target specificity. Bagher Majnooni et al. provided critical insights into combining SSs with chemotherapeutic agents and outlines key opportunities and limitations, offering valuable guidance for future anticancer drug development.

